# Common *cis* -eQTLs do not explain chromosome 21 gene expression deviations from the ploidy expectation in Trisomy 21

**DOI:** 10.64898/2026.10.10.758221

**Published:** 2026-10-13

**Authors:** Lucas A. Gillenwater, Mary A. Allen, Milton Pividori, Casey S. Greene, Marc Subirana-Granés

## Abstract

Down syndrome (DS) results from trisomy 21 (T21), the triplication of human chromosome 21 (HSA21). On average, genes on HSA21 are overexpressed at the expected 1.5-fold increase in people with DS. A small subset of HSA21 genes is expressed closer to euploid levels. Could local genetic regulatory variation explain deviations from the expected dosage? Using the largest DS-specific transcriptomic dataset to date from the Human Trisome Project (HTP) and common GTEx expression quantitative trait loci (eQTLs), we tested whether local genetic regulatory variation contributes to gene-specific buffering or amplification of HSA21 dosage effects in DS.

We compared the bulk RNA-seq profiles of protein-coding genes, lncRNAs, and pseudogenes from whole-blood samples of 274 participants with T21 and 80 euploid (D21) participants. We filtered out genes with low read counts or high genomic repeat content and applied ploidy correction to the differential expression analysis. We found 13 genes that differed significantly from the expected fold change. We cross-referenced GTEx whole-blood eQTLs ( *cis* window ±1 Mb) with the 13 deviating genes. A common *cis* -eQTL was detected for 8 of the 10 testable genes. However, 59 of 99 HSA21 genes that had the expected 1.5-fold change had significant *cis* -eQTLs. Additionally, T21 participants carry the eQTL alleles at similar frequencies to GTEx donors. Therefore, *cis* -eQTLs explain differences between people with T21, not the difference between T21 and D21.

Overall, common *cis* -regulatory variation acts on HSA21 genes in T21 as it does in euploid blood, but it does not explain which genes deviate from the ploidy expectation.

